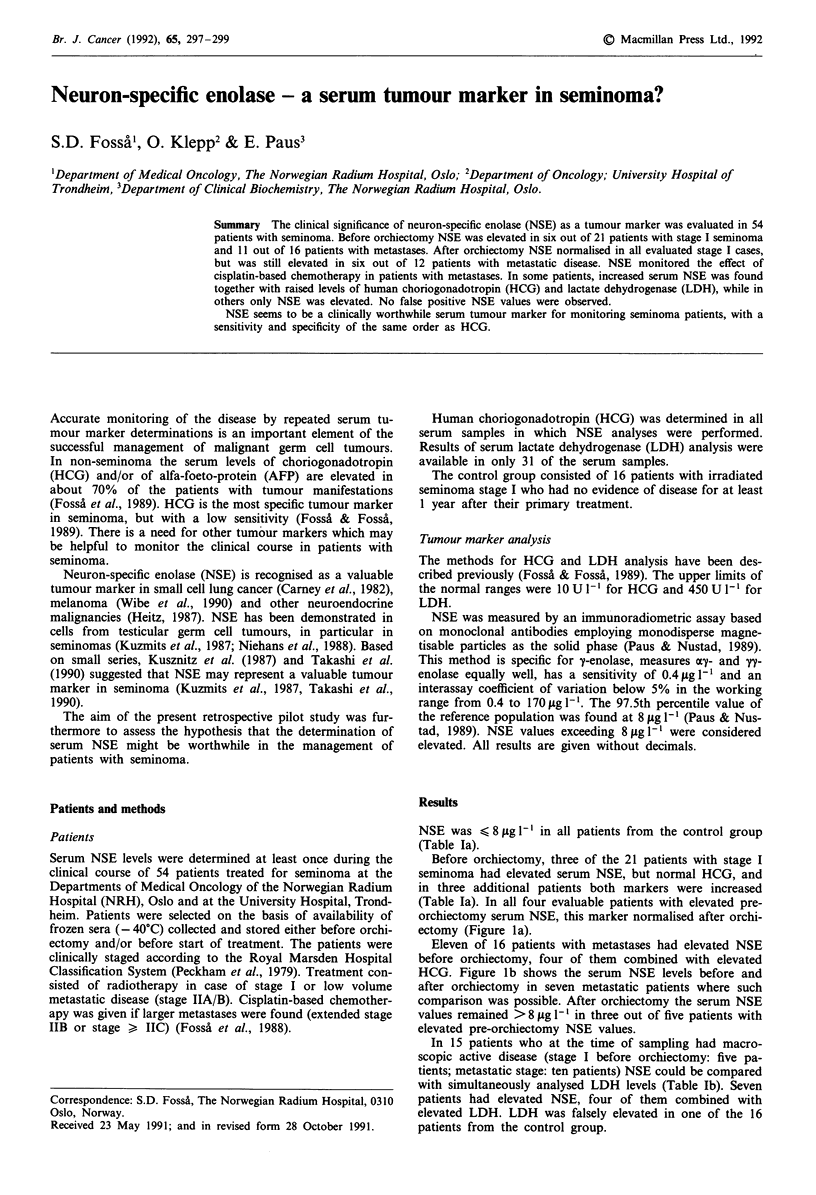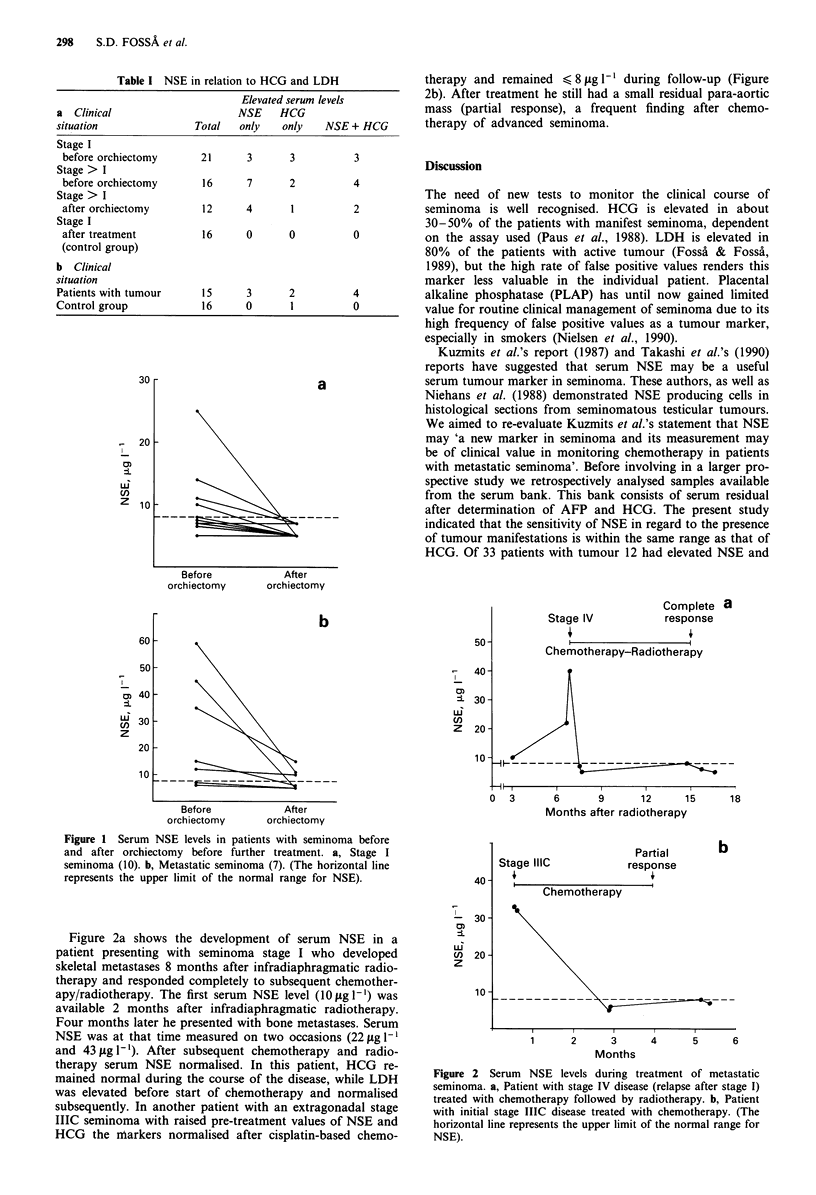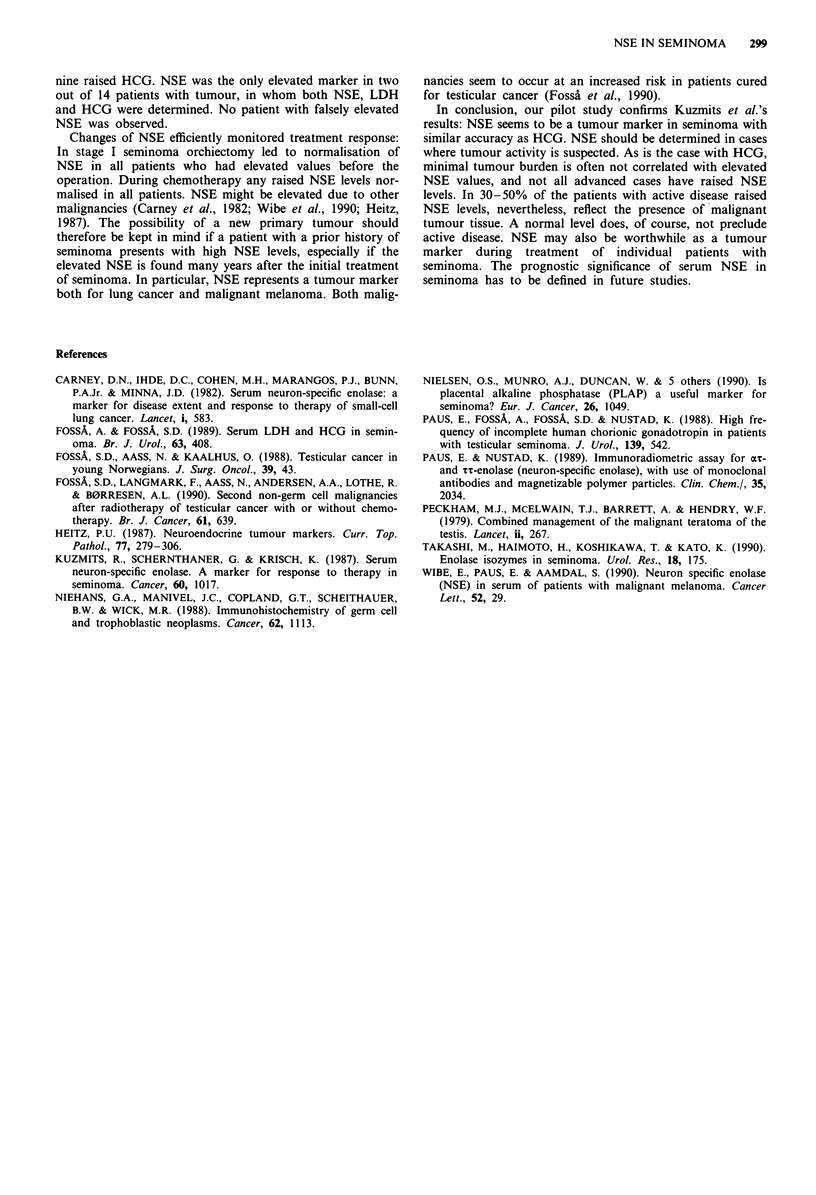# Neuron-specific enolase--a serum tumour marker in seminoma?

**DOI:** 10.1038/bjc.1992.59

**Published:** 1992-02

**Authors:** S. D. Fosså, O. Klepp, E. Paus

**Affiliations:** Department of Medical Oncology, Norwegian Radium Hospital, Oslo.

## Abstract

The clinical significance of neuron-specific enolase (NSE) as a tumour marker was evaluated in 54 patients with seminoma. Before orchiectomy NSE was elevated in six out of 21 patients with stage I seminoma and 11 out of 16 patients with metastases. After orchiectomy NSE normalised in all evaluated stage I cases, but was still elevated in six out of 12 patients with metastatic disease. NSE monitored the effect of cisplatin-based chemotherapy in patients with metastases. In some patients, increased serum NSE was found together with raised levels of human choriogonadotropin (HCG) and lactate dehydrogenase (LDH), while in others only NSE was elevated. No false positive NSE values were observed. NSE seems to be a clinically worthwhile serum tumour marker for monitoring seminoma patients, with a sensitivity and specificity of the same order as HCG.


Br. J. Cancer (1992), 65, 297–299 © Macmillan Press Ltd., 1992

### Neuron-specific enolase – a serum tumour marker in seminoma?

S.D. Fosså[1], O. Klepp[2] & E. Paus[3]

[1]*Department of Medical Oncology, The Norwegian Radium Hospital, Oslo;* [2]*Department of Oncology; University Hospital of Trondheim,* [3]*Department of Clinical Biochemistry, The Norwegian Radium Hospital, Oslo.*

**Summary** The clinical significance of neuron-specific enolase (NSE) as a tumour marker was evaluated in 54 patients with seminoma. Before orchiectomy NSE was elevated in six out of 21 patients with stage I seminoma and 11 out of 16 patients with metastases. After orchiectomy NSE normalised in all evaluated stage I cases, but was still elevated in six out of 12 patients with metastatic disease. NSE monitored the effect of cisplatin-based chemotherapy in patients with metastases. In some patients, increased serum NSE was found together with raised levels of human choriogonadotropin (HCG) and lactate dehydrogenase (LDH), while in others only NSE was elevated. No false positive NSE values were observed.

NSE seems to be a clinically worthwhile serum tumour marker for monitoring seminoma patients, with a sensitivity and specificity of the same order as HCG.

Accurate monitoring of the disease by repeated serum tumour marker determinations is an important element of the successful management of malignant germ cell tumours. In non-seminoma the serum levels of choriogonadotropin (HCG) and/or of alfa-foeto-protein (AFP) are elevated in about 70% of the patients with tumour manifestations (Fosså *et al.*, 1989). HCG is the most specific tumour marker in seminoma, but with a low sensitivity (Fosså & Fosså, 1989). There is a need for other tumour markers which may be helpful to monitor the clinical course in patients with seminoma.

Neuron-specific enolase (NSE) is recognised as a valuable tumour marker in small cell lung cancer (Carney *et al.*, 1982), melanoma (Wibe *et al.*, 1990) and other neuroendocrine malignancies (Heitz, 1987). NSE has been demonstrated in cells from testicular germ cell tumours, in particular in seminomas (Kuzmits *et al.*, 1987; Niehans *et al.*, 1988). Based on small series, Kusznitz *et al.* (1987) and Takashi *et al.* (1990) suggested that NSE may represent a valuable tumour marker in seminoma (Kuzmits *et al.*, 1987, Takashi *et al.*, 1990).

The aim of the present retrospective pilot study was furthermore to assess the hypothesis that the determination of serum NSE might be worthwhile in the management of patients with seminoma.

### Patients and methods

### *Patients*

Serum NSE levels were determined at least once during the clinical course of 54 patients treated for seminoma at the Departments of Medical Oncology of the Norwegian Radium Hospital (NRH), Oslo and at the University Hospital, Trondheim. Patients were selected on the basis of availability of frozen sera (– 40°C) collected and stored either before orchiectomy and/or before start of treatment. The patients were clinically staged according to the Royal Marsden Hospital Classification System (Peckham *et al.*, 1979). Treatment consisted of radiotherapy in case of stage I or low volume metastatic disease (stage IIA/B). Cisplatin-based chemotherapy was given if larger metastases were found (extended stage IIB or stage $\geqslant$ IIC) (Fosså *et al.*, 1988).

Correspondence: S.D. Fosså, The Norwegian Radium Hospital, 0310 Oslo, Norway.
Received 23 May 1991; and in revised form 28 October 1991.

Human choriogonadotropin (HCG) was determined in all serum samples in which NSE analyses were performed. Results of serum lactate dehydrogenase (LDH) analysis were available in only 31 of the serum samples.

The control group consisted of 16 patients with irradiated seminoma stage I who had no evidence of disease for at least 1 year after their primary treatment.

### *Tumour marker analysis*

The methods for HCG and LDH analysis have been described previously (Fosså & Fosså, 1989). The upper limits of the normal ranges were $10\,U\,l^{-1}$ for HCG and $450\,U\,l^{-1}$ for LDH.

NSE was measured by an immunoradiometric assay based on monoclonal antibodies employing monodisperse magnetisable particles as the solid phase (Paus & Nustad, 1989). This method is specific for $\gamma$-enolase, measures $\alpha\gamma$- and $\gamma\gamma$-enolase equally well, has a sensitivity of $0.4\,\mu g\,l^{-1}$ and an interassay coefficient of variation below 5% in the working range from 0.4 to $170\,\mu g\,l^{-1}$. The 97.5th percentile value of the reference population was found at $8\,\mu g\,l^{-1}$ (Paus & Nustad, 1989). NSE values exceeding $8\,\mu g\,l^{-1}$ were considered elevated. All results are given without decimals.

### Results

NSE was $\leqslant 8\,\mu g\,l^{-1}$ in all patients from the control group (Table Ia).

Before orchiectomy, three of the 21 patients with stage I seminoma had elevated serum NSE, but normal HCG, and in three additional patients both markers were increased (Table Ia). In all four evaluable patients with elevated pre-orchiectomy serum NSE, this marker normalised after orchiectomy (Figure 1a).

Eleven of 16 patients with metastases had elevated NSE before orchiectomy, four of them combined with elevated HCG. Figure 1b shows the serum NSE levels before and after orchiectomy in seven metastatic patients where such comparison was possible. After orchiectomy the serum NSE values remained $>8\,\mu g\,l^{-1}$ in three out of five patients with elevated pre-orchiectomy NSE values.

In 15 patients who at the time of sampling had macroscopic active disease (stage I before orchiectomy: five patients; metastatic stage: ten patients) NSE could be compared with simultaneously analysed LDH levels (Table Ib). Seven patients had elevated NSE, four of them combined with elevated LDH. LDH was falsely elevated in one of the 16 patients from the control group.

**Table I** NSE in relation to HCG and LDH

| | | Elevated serum levels | | |
|---|---|---|---|---|
| **a** *Clinical situation* | *Total* | *NSE only* | *HCG only* | *NSE + HCG* |
| Stage I before orchiectomy | 21 | 3 | 3 | 3 |
| Stage > I before orchiectomy | 16 | 7 | 2 | 4 |
| Stage > I after orchiectomy | 12 | 4 | 1 | 2 |
| Stage I after treatment (control group) | 16 | 0 | 0 | 0 |
| **b** *Clinical situation* | | | | |
| Patients with tumour | 15 | 3 | 2 | 4 |
| Control group | 16 | 0 | 1 | 0 |

**Figure 1** Serum NSE levels in patients with seminoma before and after orchiectomy before further treatment. **a**, Stage I seminoma (10). **b**, Metastatic seminoma (7). (The horizontal line represents the upper limit of the normal range for NSE).

Figure 2a shows the development of serum NSE in a patient presenting with seminoma stage I who developed skeletal metastases 8 months after infradiaphragmatic radiotherapy and responded completely to subsequent chemotherapy/radiotherapy. The first serum NSE level (10 $\mu g\,l^{-1}$) was available 2 months after infradiaphragmatic radiotherapy. Four months later he presented with bone metastases. Serum NSE was at that time measured on two occasions (22 $\mu g\,l^{-1}$ and 43 $\mu g\,l^{-1}$). After subsequent chemotherapy and radiotherapy serum NSE normalised. In this patient, HCG remained normal during the course of the disease, while LDH was elevated before start of chemotherapy and normalised subsequently. In another patient with an extragonadal stage IIIC seminoma with raised pre-treatment values of NSE and HCG the markers normalised after cisplatin-based chemotherapy and remained $\leqslant 8\,\mu g\,l^{-1}$ during follow-up (Figure 2b). After treatment he still had a small residual para-aortic mass (partial response), a frequent finding after chemotherapy of advanced seminoma.

### Discussion

The need of new tests to monitor the clinical course of seminoma is well recognised. HCG is elevated in about 30–50% of the patients with manifest seminoma, dependent on the assay used (Paus *et al.*, 1988). LDH is elevated in 80% of the patients with active tumour (Fosså & Fosså, 1989), but the high rate of false positive values renders this marker less valuable in the individual patient. Placental alkaline phosphatase (PLAP) has until now gained limited value for routine clinical management of seminoma due to its high frequency of false positive values as a tumour marker, especially in smokers (Nielsen *et al.*, 1990).

Kuzmits *et al.*'s report (1987) and Takashi *et al.*'s (1990) reports have suggested that serum NSE may be a useful serum tumour marker in seminoma. These authors, as well as Niehans *et al.* (1988) demonstrated NSE producing cells in histological sections from seminomatous testicular tumours. We aimed to re-evaluate Kuzmits *et al.*'s statement that NSE may 'a new marker in seminoma and its measurement may be of clinical value in monitoring chemotherapy in patients with metastatic seminoma'. Before involving in a larger prospective study we retrospectively analysed samples available from the serum bank. This bank consists of serum residual after determination of AFP and HCG. The present study indicated that the sensitivity of NSE in regard to the presence of tumour manifestations is within the same range as that of HCG. Of 33 patients with tumour 12 had elevated NSE and

**Figure 2** Serum NSE levels during treatment of metastatic seminoma. **a**, Patient with stage IV disease (relapse after stage I) treated with chemotherapy followed by radiotherapy. **b**, Patient with initial stage IIIC disease treated with chemotherapy. (The horizontal line represents the upper limit of the normal range for NSE).

nine raised HCG. NSE was the only elevated marker in two out of 14 patients with tumour, in whom both NSE, LDH and HCG were determined. No patient with falsely elevated NSE was observed.

Changes of NSE efficiently monitored treatment response: In stage I seminoma orchiectomy led to normalisation of NSE in all patients who had elevated values before the operation. During chemotherapy any raised NSE levels normalised in all patients. NSE might be elevated due to other malignancies (Carney *et al.*, 1982; Wibe *et al.*, 1990; Heitz, 1987). The possibility of a new primary tumour should therefore be kept in mind if a patient with a prior history of seminoma presents with high NSE levels, especially if the elevated NSE is found many years after the initial treatment of seminoma. In particular, NSE represents a tumour marker both for lung cancer and malignant melanoma. Both malignancies seem to occur at an increased risk in patients cured for testicular cancer (Fosså *et al.*, 1990).

In conclusion, our pilot study confirms Kuzmits *et al.*'s results: NSE seems to be a tumour marker in seminoma with similar accuracy as HCG. NSE should be determined in cases where tumour activity is suspected. As is the case with HCG, minimal tumour burden is often not correlated with elevated NSE values, and not all advanced cases have raised NSE levels. In 30–50% of the patients with active disease raised NSE levels, nevertheless, reflect the presence of malignant tumour tissue. A normal level does, of course, not preclude active disease. NSE may also be worthwhile as a tumour marker during treatment of individual patients with seminoma. The prognostic significance of serum NSE in seminoma has to be defined in future studies.

**References**

CARNEY, D.N., IHDE, D.C., COHEN, M.H., MARANGOS, P.J., BUNN, P.A.Jr. & MINNA, J.D. (1982). Serum neuron-specific enolase: a marker for disease extent and response to therapy of small-cell lung cancer. *Lancet*, **i**, 583.

FOSSÅ, A. & FOSSÅ, S.D. (1989). Serum LDH and HCG in seminoma. *Br. J. Urol.*, **63**, 408.

FOSSÅ, S.D., AASS, N. & KAALHUS, O. (1988). Testicular cancer in young Norwegians. *J. Surg. Oncol.*, **39**, 43.

FOSSÅ, S.D., LANGMARK, F., AASS, N., ANDERSEN, A.A., LOTHE, R. & BØRRESEN, A.L. (1990). Second non-germ cell malignancies after radiotherapy of testicular cancer with or without chemotherapy. *Br. J. Cancer*, **61**, 639.

HEITZ, P.U. (1987). Neuroendocrine tumour markers. *Curr. Top. Pathol.*, **77**, 279–306.

KUZMITS, R., SCHERNTHANER, G. & KRISCH, K. (1987). Serum neuron-specific enolase. A marker for response to therapy in seminoma. *Cancer*, **60**, 1017.

NIEHANS, G.A., MANIVEL, J.C., COPLAND, G.T., SCHEITHAUER, B.W. & WICK, M.R. (1988). Immunohistochemistry of germ cell and trophoblastic neoplasms. *Cancer*, **62**, 1113.

NIELSEN, O.S., MUNRO, A.J., DUNCAN, W. & 5 others (1990). Is placental alkaline phosphatase (PLAP) a useful marker for seminoma? *Eur. J. Cancer*, **26**, 1049.

PAUS, E., FOSSÅ, A., FOSSÅ, S.D. & NUSTAD, K. (1988). High frequency of incomplete human chorionic gonadotropin in patients with testicular seminoma. *J. Urol.*, **139**, 542.

PAUS, E. & NUSTAD, K. (1989). Immunoradiometric assay for ατ- and ττ-enolase (neuron-specific enolase), with use of monoclonal antibodies and magnetizable polymer particles. *Clin. Chem.*/, **35**, 2034.

PECKHAM, M.J., MCELWAIN, T.J., BARRETT, A. & HENDRY, W.F. (1979). Combined management of the malignant teratoma of the testis. *Lancet*, **ii**, 267.

TAKASHI, M., HAIMOTO, H., KOSHIKAWA, T. & KATO, K. (1990). Enolase isozymes in seminoma. *Urol. Res.*, **18**, 175.

WIBE, E., PAUS, E. & AAMDAL, S. (1990). Neuron specific enolase (NSE) in serum of patients with malignant melanoma. *Cancer Lett.*, **52**, 29.